# Phosphoprotein enriched in astrocytes (PEA)-15: A potential therapeutic target in multiple disease states

**DOI:** 10.1016/j.pharmthera.2014.03.006

**Published:** 2014-09

**Authors:** Fiona H. Greig, Graeme F. Nixon

**Affiliations:** School of Medical Sciences, University of Aberdeen, Foresterhill, Aberdeen AB25 2ZD, UK

**Keywords:** PEA-15, ERK1/2, Proliferation, Apoptosis, Cancer, Type 2 diabetes, CaMKII, calcium/calmodulin-dependent protein kinase II, DED, death effector domain, DISC, death initiation signalling complex, ERK1/2, extracellular signal-regulated kinases 1/2, FADD, Fas-associated death domain protein, GLUT, glucose transporter, HNF-4α, hepatocyte nuclear factor 4alpha, IL, interleukin, MAP, mitogen-activated protein, PEA-15, phosphoprotein enriched in astrocytes-15, PCOS, polycystic ovary syndrome, PDGF, platelet-derived growth factor, PKC, protein kinase C, PLD1, phospholipase D1, NSCLC, non-small cell lung cancer, RSK, ribosomal s6 kinase, TGF-β1, transforming growth factor-β1, TRAIL, tumour necrosis factor-related apoptosis-inducing ligand, VSM, vascular smooth muscle

## Abstract

Phosphoprotein enriched in astrocytes-15 (PEA-15) is a cytoplasmic protein that sits at an important junction in intracellular signalling and can regulate diverse cellular processes, such as proliferation and apoptosis, dependent upon stimulation. Regulation of these processes occurs by virtue of the unique interaction of PEA-15 with other signalling proteins. PEA-15 acts as a cytoplasmic tether for the mitogen-activated protein kinases, extracellular signal-regulated kinase 1/2 (ERK1/2) preventing nuclear localisation. In order to release ERK1/2, PEA-15 requires to be phosphorylated via several potential pathways. PEA-15 (and its phosphorylation state) therefore regulates many ERK1/2-dependent processes, including proliferation, via regulating ERK1/2 nuclear translocation. In addition, PEA-15 contains a death effector domain (DED) which allows interaction with other DED-containing proteins. PEA-15 can bind the DED-containing apoptotic adaptor molecule, Fas-associated death domain protein (FADD) which is also dependent on the phosphorylation status of PEA-15. PEA-15 binding of FADD can inhibit apoptosis as bound FADD cannot participate in the assembly of apoptotic signalling complexes. Through these protein–protein interactions, PEA-15-regulated cellular effects have now been investigated in a number of disease-related studies. Changes in PEA-15 expression and regulation have been observed in diabetes mellitus, cancer, neurological disorders and the cardiovascular system. These changes have been suggested to contribute to the pathology related to each of these disease states. As such, new therapeutic targets based around PEA-15 and its associated interactions are now being uncovered and could provide novel avenues for treatment strategies in multiple diseases.

## 1 Introduction

Since the initial characterisation of phosphoprotein enriched in astrocytes-15 (PEA-15) almost 20 years ago (Araujo et al., 1993, Danziger et al., 1995), the unique function of this protein in regulating intracellular pathways has become increasingly evident. Sitting at a critical signalling junction which regulates cell behaviour, PEA-15 has the potential to direct cell phenotypic modulation (Renault et al., 2003, Valmiki and Ramos, 2009). Recent studies, both biochemical and structural, have begun to further tease out the molecular mechanisms of these protein interactions in greater detail and led to an enhanced understanding of the biology of PEA-15 and its important role in many cellular processes. Significant evidence has also indicated the involvement of PEA-15 and PEA-15-regulated intracellular signalling in several different diseases including cancer, diabetes mellitus, neurological disorders and cardiovascular disease. PEA-15 therefore represents a novel and therapeutically relevant target. This review will examine, in detail, current findings with regard to PEA-15's intracellular functions. In addition, it will evaluate the potential role of PEA-15 in specific disease states with a view to identifying new areas for therapeutic intervention.

## 2 Structure and signalling of phosphoprotein enriched in astrocytes-15

PEA-15 was originally identified in primary cultured astrocytes following a search for phosphoproteins which might regulate astrocyte function (Araujo et al., 1993). In this initial study, two phosphorylation sites were identified. Subsequent studies demonstrated that these sites are Ser^104^ and Ser^116^ and are preferentially phosphorylated by either protein kinase C (PKC) at Ser^104^ or calcium/calmodulin-dependent protein kinase II (CaMKII) or Akt at Ser^116^ (Araujo et al., 1993, Estelles et al., 1996, Kubes et al., 1998). A further study revealed that PEA-15 contained a death effector domain (DED), typically associated with proteins involved in the apoptotic cascade, and provided the first functional evidence of a cellular role for PEA-15 (Ramos et al., 1998). This role was indicated by its ability to block the extracellular signal-regulated kinases 1/2 (ERK1/2)-mediated H-Ras suppression of integrin activation (Ramos et al., 1998). The DED was required for this blockade but PEA-15 did not prevent ERK1/2 activation by Ras. This was one of the first examples of a DED-containing protein regulating processes other than apoptosis. Since these initial studies, multiple investigations have followed and important details of the structure and function of PEA-15 have now been uncovered.

One of the major cellular functions of PEA-15 is as an ERK1/2 binding protein (Formstecher et al., 2001, Hill et al., 2002). ERK1 and ERK2 (conventionally treated as the same enzyme, ERK1/2, due to a high sequence homology) are members of the mitogen-activated protein (MAP) kinase family and are involved in many critical aspects of cell signalling (Ramos, 2008). Regulation of ERK1/2 is via a canonical cascade involving several signalling components resulting in the activation of the MAP kinase kinase, MEK1/2, which phosphorylates ERK1/2 on both serine and threonine residues. This phosphorylation activates ERK1/2 and results in a wide range of signalling outcomes including cytoplasmic interactions (such as with the MAP kinase effector, ribosomal s6 kinase (RSK)) and, following translocation to nucleus, interaction with the ETS transcription factor, Elk-1 which is an important effector for ERK1/2-mediated cellular effects such as proliferation (Buchwalter et al., 2004). The interaction of ERK1/2 with its multiple substrates occurs via two docking domains; the D-domain (common in MAP kinases) and a *d*ocking site for *E*RK, *F*XFP (DEF) motif (Roskoski, 2012). As these enzymes are critical in many different cellular processes, it is perhaps not surprising that the regulation of ERK1/2 activation (upstream effects) and the consequences of this activation (downstream actions) are complex and involve multiple components. These include several scaffolding proteins which can influence ERK1/2 activation, e.g. kinase suppressor of Ras (Yu et al., 1998). Within this complex regulatory signal, studies have now shown that PEA-15, via its ERK1/2 binding properties, is likely to have a unique role as a downstream regulator of the intracellular effects of ERK1/2.

**Fig. 1:**
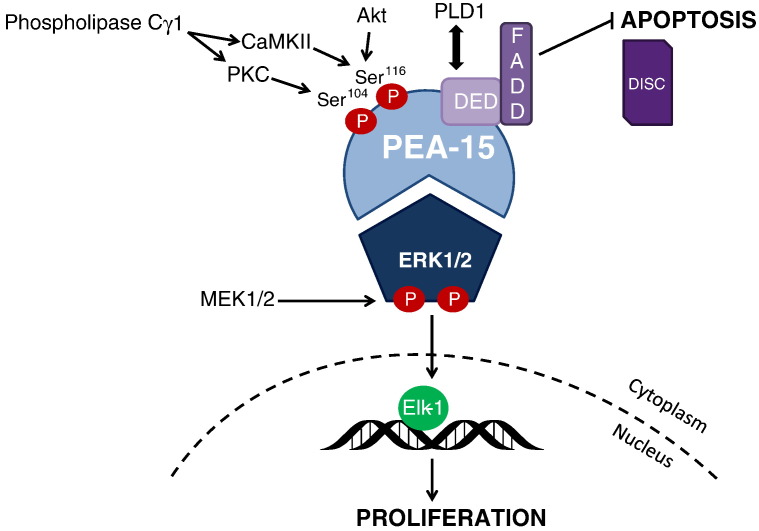
The role of PEA-15 in proliferation and apoptosis. PEA-15 has two phosphorylation sites which are preferentially phosphorylated by PKC at Ser^104^ and CaMKII or Akt at Ser^116^. PKC and CaMKII can both be activated by phospholipase Cγ1. PEA-15 is an ERK1/2 binding protein and acts as an “anchor” of ERK1/2 by sequestering it in the cytoplasm, preventing subsequent translocation into the nucleus. PEA-15 can bind both ERK1/2 and phosphorylated ERK1/2 with equal affinity which is activated by the upstream MAP kinase kinase, MEK1/2. Phosphorylation of PEA-15 releases ERK1/2 resulting in the activation of the nuclear transcription factor, Elk-1 and proliferation of the cell. PEA-15 contains a DED which upon phosphorylation promotes the binding of FADD. The association of PEA-15 and FADD prevents FADD-mediated activation of caspases and the formation of the DISC resulting in an inhibition of apoptosis. The DED of PEA-15 also interacts with PLD1.

Substantial evidence now demonstrates that PEA-15 acts as a cytoplasmic “anchor” for ERK1/2 (Formstecher et al., 2001). PEA-15 signalling and its association with ERK1/2 are illustrated in Fig. 1. By binding to ERK1/2 and sequestering it in the cytoplasm, PEA-15 regulates the outcome of ERK1/2 signalling. Although PEA-15 can enter the nucleus in limited circumstances for a short time period, it is almost exclusively confined to the cytoplasm as it contains a nuclear export sequence (Formstecher et al., 2001). The role of PEA-15 in determining ERK1/2 localisation, and therefore ERK1/2 signalling outcomes, has now been demonstrated in a number of different cell types including astrocytes (Formstecher et al., 2001, Sharif et al., 2003), fibroblasts (Gaumont-Leclerc et al., 2004, Buonomo et al., 2012), testicular sertoli cells (Mizrak et al., 2007), T-lymphocytes (Pastorino et al., 2010), pituitary gonadotrope cells (Choi et al., 2011) and vascular smooth muscle (VSM) cells (Hunter et al., 2011). In all these cell types, PEA-15 regulation of ERK1/2 has major effects on cell function with implications for the respective organ systems involved. Cellular overexpression of PEA-15 leads to decreased ERK1/2 nuclear translocation resulting in the predicted decreased activation of nuclear substrates of ERK1/2 such as phosphorylation of Elk-1 (Formstecher et al., 2001). Altering PEA-15 expression levels does not affect the activation of ERK1/2 (non-nuclear) cytoplasmic substrates such as RSK. In fact, PEA-15 acts as a RSK scaffold facilitating RSK activation by ERK1/2 (Vaidyanathan et al., 2007). Converse effects on ERK1/2 localisation occur when PEA-15 expression is knocked down using an RNAi or gene deletion, producing an increase in ERK1/2 nuclear localisation (Formstecher et al., 2001, Pastorino et al., 2010, Choi et al., 2011, Hunter et al., 2011).

Importantly, PEA-15 is not an upstream regulator and does not alter the ability of MEK1/2 to activate ERK1/2 but can directly block ERK1/2 interaction with downstream mediators (Formstecher et al., 2001, Callaway et al., 2007). Both ERK1/2 and phosphorylated ERK1/2 bind with equal affinity to PEA-15 (Callaway et al., 2007). Recent studies have now revealed further details about the binding interaction between ERK1/2 and PEA-15. PEA-15 binds to ERK1/2 via a bipartite mode which leads to a tight interaction of these two proteins (Mace et al., 2013). This includes both the DED and a region within the C terminus of PEA-15 (Hill et al., 2002, Farina et al., 2010, Twomey and Wei, 2012, Mace et al., 2013). Through this enhanced bipartite binding, ERK1/2 is tightly coupled and, despite ERK1/2 phosphorylation, ERK1/2 cannot activate at least some downstream substrates (Callaway et al., 2007). This is due to PEA-15 binding which blocks the D-peptide-binding site (or D-recruitment site), the site required for interaction with ERK substrates. Solving the crystal structures of PEA-15 bound to ERK2 has revealed further interesting details. Analyses of these structures revealed that PEA-15 actually protects ERK1/2 from dephosphorylation and in fact acts as a “primer” to hold activated ERK1/2 in a state which is readied for downstream signalling once ERK1/2 is released from the PEA-15 complex (Mace et al., 2013). It appears, therefore, that PEA-15 is not merely a scaffold protein for ERK1/2 localisation but in fact an integral mechanism which can directly regulate ERK1/2 signalling independent of the recognised canonical activation pathway. This puts PEA-15 in a unique position in terms of controlling cell function and suggests that merely assessing ERK1/2 activation as an indicator of the well-described ERK1/2-mediated outcomes, such as proliferation, is insufficient.

As PEA-15 is tightly bound to ERK1/2 preventing further downstream signalling (but not affecting ERK1/2 activation), the release mechanisms from this inhibitory complex are therefore critical regulatory components. Several reports have demonstrated that the release mechanism is in fact the phosphorylation of PEA-15 at Ser^104^ (phosphorylated by PKC) and Ser^116^ sites (phosphorylated by CaMKII or Akt) (Trencia et al., 2003, Krueger et al., 2005, Renganathan et al., 2005). Phosphorylation of PEA-15 at least at Ser^104^ blocks ERK1/2 binding, though phosphorylation of both sites is possibly required depending upon cell type. PEA-15 phosphorylation leads to an increase in the nuclear localisation of ERK1/2 and activation of nuclear transcription (Krueger et al., 2005, Renganathan et al., 2005). Using anisotropy methods, one study however suggests that the affinity of PEA-15 for ERK1/2 in vitro is not altered by PEA-15 phosphorylation (Callaway et al., 2007). The exact mechanism of the release of ERK1/2 is therefore not completely defined although it is clear that changes in PEA-15 phosphorylation can alter the physiological outcomes of ERK1/2 signalling (Krueger et al., 2005, Renganathan et al., 2005). While further evidence is awaited on the exact nature of the release mechanisms, it does appear that in terms of ERK1/2 signalling, PEA-15 is the “trigger” which, when released by phosphorylation, allows ERK1/2 to translocate to the nucleus and regulate transcription. If this is indeed the mechanism involved, an interesting scenario is suggested whereby regulation of two (potentially distinct) pathways may be required to allow for ERK1/2 signalling; one pathway to stimulate the canonical activation of ERK1/2 and another to phosphorylate PEA-15. Indeed, we have shown that knockdown of phospholipase Cγ1 (thereby inhibiting activation of both PKC and CaMKII) prevents the platelet-derived growth factor (PDGF)-induced translocation of ERK1/2 (and the subsequent activation of Elk-1) by blocking PEA-15 phosphorylation but does not prevent ERK1/2 activation (Hunter et al., 2011). In terms of ERK1/2 transcription requiring ERK1/2 translocation to the nucleus, consideration of PEA-15 phosphorylation could therefore be of equal importance to the ERK1/2 upstream cascade.

While the phosphorylation of PEA-15 probably acts as a trigger-release mechanism for ERK1/2-mediated cellular outcomes, this phosphorylation also modulates other non-ERK1/2 signalling pathways. Specifically, PEA-15 phosphorylation at Ser^116^ by CaMKII or Akt promotes the binding of Fas-associated death domain protein (FADD) to the DED domain of PEA-15 (Condorelli et al., 1999, Kitsberg et al., 1999). FADD is an essential part of death initiation signalling complex (DISC) which assembles proteins involved in the apoptotic process such as caspases (Valmiki & Ramos, 2009). The interaction between PEA-15 and FADD blocks apoptosis as it prevents FADD-directed activation of caspases in the DISC (Condorelli et al., 1999, Xiao et al., 2002, Renganathan et al., 2005, Peacock et al., 2009). Phosphorylated PEA-15 itself can be recruited to the DISC during this process (Xiao et al., 2002). This anti-apoptotic role of PEA-15 is observed in astrocytes isolated from PEA-15 null mice which revealed an increased apoptosis following incubation with tumour necrosis factor alpha (Kitsberg et al., 1999). T-cells from these same mice, however, did not have altered apoptotic responses to pro-apoptotic stimuli (Pastorino et al., 2010). This may represent a level of cell-type specificity. Regardless, changing the phosphorylation level of PEA-15 does appear to have an important role in switching cells into an anti-apoptotic state in several of the cell types studied to date (Sulzmaier et al., 2012a). Such changes to cell phenotype have obvious implications in cancer and these will be explored in greater detail later in this review.

A further protein–protein interaction with PEA-15 has been observed which can have effects on cell function and may be important in some disease states. PEA-15 binds and interacts with the phospholipase D1 (PLD1), which hydrolyses phosphatidylcholine into the important mediator, phosphatidic acid (Zhang et al., 2000). This binding occurs in the N-terminal of PEA-15 which contains a D-peptide-binding motif targeted by protein which contain a D-motif including enzymes such as PLD1 (Viparelli et al., 2008). This interaction is not dependent on the phosphorylation state of PEA-15 (Sulzmaier et al., 2012b). Studies have demonstrated that PLD1 activity is increased when PEA-15 is present, and this is via elevated PLD1 expression (a potential effect on increased PLD1 protein stability) (Zhang et al., 2000). Such an increase could lead to enhanced downstream effects due to increased levels of phosphatidic acid, for example activation of ERK1/2 via phosphatidic acid-induced elevation of upstream activators of the ERK1/2 cascade or increased production of diacylglycerol which activates PKC isoforms (Besterman et al., 1986, Rizzo et al., 2000). This may ultimately alter cell phenotype through regulation of proliferation (Sulzmaier et al., 2012a). The PEA-15 interaction with PLD1 can also inhibit insulin-dependent glucose uptake in a skeletal muscle cell line when PEA-15 is overexpressed (Viparelli et al., 2008).

**Fig. 2:**
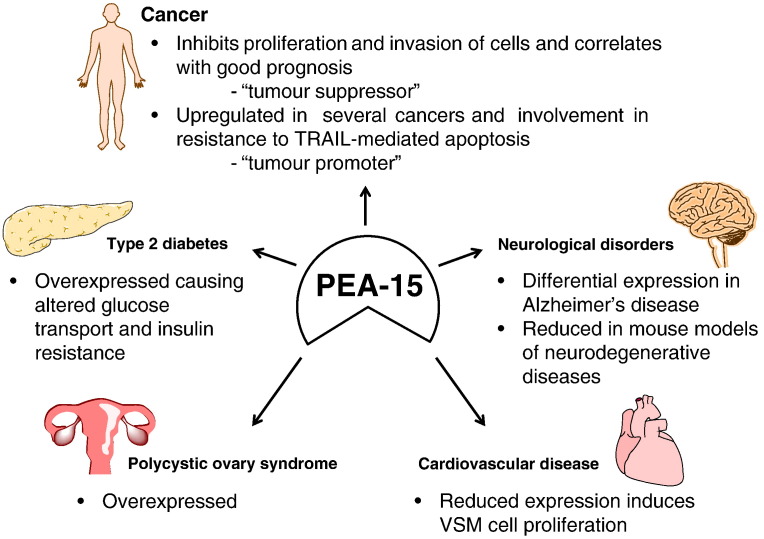
PEA-15 in multiple disease states. PEA-15 acts as both a tumour suppressor and a tumour promoter in cancer by inhibiting proliferation and invasion of cancerous cells and mediating resistance to TRAIL-induced apoptosis, respectively. PEA-15 is also overexpressed in type 2 diabetes and PCOS causing altered glucose transport. Reduced PEA-15 expression inhibits VSM cell proliferation in cardiovascular disease and the levels of PEA-15 are also reduced in various neurodegenerative diseases.

As PEA-15 is centrally located within the intracellular signalling landscape, it can be readily appreciated that this protein could potentially be involved in several different disease states. With regulation in proliferation and apoptosis, in addition to interaction with several critical proteins including ERK1/2, it is not surprising that a major role of PEA-15 in several diseases is beginning to emerge, summarised in Fig. 2. This includes different types of cancer where the balance of proliferation and apoptosis is critical. PEA-15 is overexpressed in diabetes mellitus and can regulate insulin sensitivity and glucose uptake. Differential expression of PEA-15 in the brain has also been associated with neurological disorders such as Alzheimer's and Huntington's diseases. In cardiovascular disease, our recent study has indicated that PEA-15 may regulate VSM cell proliferation and could therefore be involved in restenosis following angioplasty in the treatment of atherosclerosis. Involvement in different aspects of all these disease states raises the potential of PEA-15 being an effective therapeutic target or prognostic tool which will be explored throughout this review.

## 3 Phosphoprotein enriched in astrocytes-15 in cancer

Abnormal cellular signalling is fundamental in cancer progression and tumourigenesis. PEA-15 was implicated in the dysregulation of these pathological processes due to its intracellular function. PEA-15 has now been described as both tumour suppressor and promoter due to its inhibition of proliferation and invasion of some cancerous cells (Glading et al., 2007, Bartholomeusz et al., 2008, Gawecka et al., 2012) as well as its upregulation and involvement in resistance to tumour necrosis factor-related apoptosis-inducing ligand (TRAIL)-mediated apoptosis in several cancer types (Stassi et al., 2005, Garofalo et al., 2007, Heikaus et al., 2008, Zanca et al., 2008). This reveals the potential for PEA-15 to be utilised as either a prognostic marker or a therapeutic target in specific cancer subsets which could be dependent, in part, on the phosphorylation status of the protein (Sulzmaier et al., 2012a).

Cancer is characterised by unregulated cell growth resulting in the formation of malignant tumours which can invade and destroy surrounding healthy tissue and can also metastasise in other areas of the body. Normal quiescent cells transform into active proliferative cancerous cells with several acquired capabilities including the generation of their own growth stimuli resulting in a limitless replicative potential, resistance to anti-growth signals, apoptosis and destruction by the immune system, angiogenetic properties, reprogramming of energy metabolism and the ability to invade adjacent tissue resulting in tumour formation and metastasis (Hanahan and Weinberg, 2000, Hanahan and Weinberg, 2011). Genetic components, environmental factors and lifestyle choices such as smoking and dietary intake all contribute to the pathogenesis of cancer causing mutations that produce either oncogenes with a dominant gain of function or tumour suppressor genes with a recessive loss of function (Hanahan & Weinberg, 2000). With over 200 different types of cancer affecting the population worldwide, there is a vital need for a greater understanding of the disease progression which could result in novel therapeutic strategies to combat the global burden of cancer.

### 3.1 Phosphoprotein enriched in astrocytes-15 as a tumour suppressor

Regulation of the ERK1/2 signalling cascade by PEA-15 has been associated with several beneficial effects on inhibiting tumour development. The adenovirus type 5 gene, *E1A* is known to suppress tumourigenecity and inhibit overexpression of HER2 in breast and ovarian cancers (Yu et al., 1993, Chang et al., 1997). PEA-15 is upregulated by *E1A* resulting in reduced proliferation in ovarian cancer by inhibition of ERK1/2-dependent transcription (Bartholomeusz et al., 2006). PEA-15 also inhibited migration and invasion of cells in breast cancer by its interaction with ERK1/2 resulting in sequestering of ERK1/2 in the cytoplasm and preventing nuclear translocation (Glading et al., 2007). Moreover, PEA-15 blocked tumourigenesis in a triple-negative breast cancer xenograft model by an ERK1/2-dependent mechanism with overexpression of PEA-15 resulting in elevated caspase-8-dependent apoptosis (Bartholomeusz et al., 2010). In glioblastoma cultures, migrating cells from tumour explants expressed low levels of PEA-15 and this inhibitory control of PEA-15 on cell motility was through a PKCδ-dependent mechanism (Renault-Mihara et al., 2006). PEA-15 was also shown to induce cellular senescence in human fibroblasts preventing their transformation (Gaumont-Leclerc et al., 2004).

In addition to impairment of cell migration and proliferation, PEA-15 expression correlates with good prognosis in neuroblastoma and a 25% increase in patient survival time with the highest PEA-15 levels found in early stage tumours (Gawecka et al., 2012). In astrocytoma, PEA-15 levels were inversely associated with the stage of the tumour (Watanabe et al., 2010). There is also potential for PEA-15 to be used as a prognostic tool in ovarian cancer as women with tumours expressing high levels of PEA-15 survived for longer (Bartholomeusz et al., 2008). PEA-15 which was unphosphorylated at both sites significantly inhibited migration as well as angiogenesis in vivo which was partially dependent on β-catenin expression (Lee et al., 2012). This would suggest the potential for PEA-15 as a tumour suppressor and prognostic marker in cancer with the phosphorylation status of PEA-15 thought to be influential in regulating the function of PEA-15 (Sulzmaier et al., 2012a). However in a recent study, bisphosphorylated PEA-15 was also shown to sensitise ovarian cancer cells to the chemotherapeutic agent, paclitaxel by promoting apoptosis via impairment of the microtubule-destabilising effect of SCLIP, a SCG10-like protein (Xie et al., 2013).

### 3.2 Phosphoprotein enriched in astrocytes-15 as a tumour promoter

In contrast to its tumour suppressing function, there is now mounting evidence for an oncogenic role of PEA-15 in several cancer types. PEA-15 is known to be upregulated in a variety of cancer subsets including immortal cancer cell lines (e.g. MCF-7 and HeLa cells) (Condorelli et al., 1999), malignant pleural mesothelioma cells (Kuramitsu et al., 2009), breast cancer cells (Stassi et al., 2005), non-small cell lung cancer (NSCLC) cells (Zanca et al., 2008), glioblastoma (Eramo et al., 2005) and renal cell carcinomas (Heikaus et al., 2008). Mice overexpressing PEA-15 also displayed an increase in skin tumourigenesis with a four-fold increase in papilloma number compared to their wild type littermates (Formisano et al., 2005), suggesting a potential role for PEA-15 in tumour formation and cancer progression.

In NSCLC, PEA-15 has been shown to interact with Rac1, a Rho GTPase, and aids in Rac1 activation resulting in modulation of migration and invasion (Zanca et al., 2010). PEA-15 also regulates cellular invasion in colorectal carcinomas and was observed in well differentiated tumour areas (Funke et al., 2013). PEA-15 interaction with the 67 kDa laminin receptor (67LR), which has been suggested as a marker for metastasis (Menard et al., 1998), significantly increased 67LR-mediated migration and proliferation (Formisano et al., 2012). Furthermore, when the proto-oncogene, H-Ras, is constitutively active, PEA-15 was found to potentiate ERK1/2 signalling and enhance H-Ras-driven transformation of kidney epithelial cells which was mediated, in part, by its interaction with PLD1 (Sulzmaier et al., 2012b). This highlights an involvement of PEA-15 in many of the cellular processes which are critical in tumourigenesis. High ERK1/2 levels were found to correlate with PEA-15 expression and shorter survival times in patients with triple-negative breast cancer (Bartholomeusz et al., 2012).

Alterations in apoptotic signalling have also been identified in several cancers with changes in expression of apoptosis-related genes (Debatin & Krammer, 2004). TRAIL preferentially induces apoptosis in tumour cells opposed to normal healthy cells, suggesting a use as a tumour-specific chemotherapeutic agent (Kim et al., 2000). The anti-apoptotic protein, PEA-15 has been implicated in TRAIL resistance as well as insensitivity to other forms of apoptosis in several cancer types resulting in increased cell survival and tumour formation. Increased levels of PEA-15 were observed in glioblastoma, lymphocytes of patients with B-cell chronic lymphocytic leukaemia and in tumours of NSCLC patients; all of which are resistant to TRAIL (Eramo et al., 2005, Garofalo et al., 2007, Zanca et al., 2008). Similarly, glioma cell lines resistant to TRAIL were found to have a two-fold increase in PEA-15 expression and inhibition of PKC re-established sensitivity to TRAIL, implicating phosphorylation of PEA-15 in this process (Hao et al., 2001). TRAIL has four membrane-bound cell surface receptors to regulate the apoptotic response (Kim et al., 2000). One of these receptors, death receptor 5 (DR5) was consistently expressed in glioblastoma cell lines and tumour samples and when activated by TRAIL, recruited FADD and caspase-8 for the assembly of DISC (Bellail et al., 2010). However, DR5-mediated DISC was modified by several anti-apoptotic proteins including PEA-15 resulting in TRAIL resistance in glioblastoma (Bellail et al., 2010). Recently, microRNA-212 has been suggested to be a negative regulator of PEA-15 in NSCLC as overexpression of the microRNA resulting in a reduction in PEA-15 protein levels and increased TRAIL-mediated apoptosis, suggesting they are inversely correlated (Incoronato et al., 2010).

In addition to TRAIL resistance, PEA-15 has been implicated in other mechanisms of cell death. In thyroid cancer, PEA-15 is upregulated by the cytokines, interleukin (IL)-4 and -10 which causes resistance to CD95 or Fas activation (Todaro et al., 2006). IL-4 induced the overexpression of PEA-15 and other anti-apoptotic proteins in epithelial cancers such as colon, breast and lung carcinomas resulting in resistance to apoptosis (Todaro et al., 2008). Upregulation of PEA-15 also led to an increase in resistance to glucose deprivation-induced apoptosis of glioblastoma cells and an increase in phosphorylation at Ser^116^ of PEA-15 was evident in perinecrotic areas (Eckert et al., 2008). However, protein phosphatase 4-induced apoptosis of human T-cells, which are important in preventing leukaemia and lymphoma, was found to be partly mediated by PEA-15 dephosphorylation (Mourtada-Maarabouni & Williams, 2009).

Pharmacologically targeting the resistance to apoptotic signalling in cancerous cells could potentially reduce tumour formation. Treatment with the flavonoid, epigalocatechin-3-gallate, in glioblastoma cells caused a downregulation in PEA-15 expression through an Akt (PKB)-dependent mechanism and in combination with TRAIL could be used to induce apoptosis in tumour cells (Siegelin et al., 2008). The protective role of PEA-15 on glucose withdrawal was abolished in glioma cells treated with a MEK1/2 inhibitor suggesting that the pro-survival effects are mediated through ERK1/2 signalling and could be exploited to help combat resistance to apoptotic mechanisms (Eckert et al., 2008). In addition, in prostate cancer cell lines resistant to TRAIL, incubation with the chemotherapy drug, decitabine, sensitises these cells to TRAIL-mediated apoptosis, partly through a reduction in anti-apoptotic proteins including PEA-15 (Festuccia et al., 2008). Microarray analysis of liposarcoma cultures after treatment with doxorubicin, a potent chemotherapeutic agent with a limited success rate, was shown to upregulate several pro-apoptotic proteins; however, some anti-apoptotic proteins were also upregulated such as PEA-15 (Daigeler et al., 2008).

Overall, this provides evidence for the role of PEA-15 as both tumour suppressor and tumour promoter in cancer. Sulzmaier and colleagues suggested that the switch between these two states was due to the phosphorylation status of PEA-15 with unphosphorylated PEA-15 demonstrating tumour suppressor qualities while phosphorylation of PEA-15 induced higher tumour cell survival and resistance to apoptosis. However, a recent study demonstrated that phosphorylated PEA-15 sensitised ovarian cancer cells to a chemotherapeutic agent (Xie et al., 2013), suggesting that further investigation of the effect of phosphorylation of PEA-15 in inhibiting tumour development might be warranted.

## 4 Phosphoprotein enriched in astrocytes-15 in diseases of the endocrine system

Diseases of the endocrine system are often extremely complex due to dysregulation of negative feedback mechanisms to maintain homeostasis resulting in either hypo- or hypersecretion of hormones or a combination of both. The most common endocrine disease is diabetes mellitus affecting glucose homeostasis where 10% of people have type 1 diabetes while 90% of those diagnosed have type 2 diabetes. In addition, polycystic ovary syndrome (PCOS) is one of the most prevalent endocrine disorders in women, affecting around 1 in 15 worldwide (Norman et al., 2007). This suggests a mounting need for novel therapeutic targets to combat the increasing occurrence of these endocrinological disorders within the population.

### 4.1 Phosphoprotein enriched in astrocytes-15 in type 2 diabetes

Type 2 diabetes is a complex heterogeneous metabolic disorder which is characterised by chronic hyperglycaemia. The elevated levels of serum glucose are a consequence of deficiencies in both insulin secretion and sensitivity. Pancreatic β-cell dysfunction results in an impaired insulin regulation of glucose transport and therefore reduced insulin secretion which is necessary for disease progression (Efendic & Ostenson, 1993). The major sites of insulin resistance are the liver and extrahepatic tissues, primarily skeletal muscle and adipose tissue (DeFronzo et al., 1992, Gerich, 1998). The pathogenesis of type 2 diabetes results from the interaction of genetic and environmental components such as obesity, age and lifestyle. Type 2 diabetes displays polygenic inheritance; however, the gene or genes responsible for this predisposition are currently unknown. Random genome-wide screens were utilised in an effort to locate these genes and identified potential susceptible loci in different ethnic groups (Hanis et al., 1996, Mahtani et al., 1996). Mutations in the insulin signalling pathway and mitochondrial DNA were also found in diabetic individuals (Kishimoto et al., 1992, Reardon et al., 1992, Kahn, 1994); however, all of these abnormalities in gene expression account for only a small proportion of patients.

Overexpression of the gene encoding PEA-15 in skeletal muscle and adipose tissue of type 2 diabetic patients, major sites of insulin resistance, was revealed by gene expression studies (Condorelli et al., 1998). This also resulted in the alternative name for PEA-15 of phosphoprotein enriched in diabetes or PED. Healthy first-degree relatives of type 2 diabetic patients had two-fold higher levels of PEA-15 expression compared with individuals with no family history of the disease (Valentino et al., 2006). This effect was also inversely correlated with insulin sensitivity and independent of sex, age and other dietary conditions (Valentino et al., 2006). Furthermore, offspring of type 2 diabetic patients display a significant genetic risk of developing the disease (Klein et al., 1996). Mice overexpressing the *ped/pea-15* gene were also found to develop insulin resistance as following insulin administration, a reduction of only 35% in the plasma glucose levels was observed after 45 min compared to 70% in control mice (Vigliotta et al., 2004). Cloning of the promoter region of the human *pea-15* gene revealed a cis-acting regulatory element which binds hepatocyte nuclear factor 4alpha (HNF-4α) which is involved in glucose and lipid homeostasis (Ungaro et al., 2008). HNF-4α repressed PEA-15 expression in HeLa cells and HNF-4α silencing correlated with PEA-15 overexpression resulting in a significant reduction in hepatic glycogen content which could account for the elevated expression levels witnessed in type 2 diabetes (Ungaro et al., 2008, Ungaro et al., 2010). These findings highlight the potential significance of PEA-15 at the initial onset and also during disease progression in both mouse models and type 2 diabetic patients.

Insulin signalling and glucose transport are critical in maintaining glucose homeostasis and defects in these pathways significantly contribute to the pathogenesis of type 2 diabetes. PEA-15 was reported to alter the expression of two of the major glucose transporter isoforms, GLUT1 and GLUT4, in both skeletal muscle and adipocytes (Condorelli et al., 1998). Overexpression of PEA-15 resulted in increased expression of GLUT1 at the plasma membrane which prevented additional membrane translocation of the transporter after insulin stimulation and also further inhibited insulin-dependent glucose transport by blocking GLUT4 membrane recruitment (Condorelli et al., 1998). This higher basal level of glucose uptake in cells overexpressing PEA-15 impairs further insulin action therefore leading to insulin resistance. This is accompanied by the PLD-dependent stimulation of conventional PKC isoform, PKCα by PEA-15 which is constitutively active in these cells and prevents subsequent activation of the atypical PKCζ by insulin (Condorelli et al., 1998, Condorelli et al., 2001). The PKCζ isoform is a primary activator of GLUT4 translocation to the plasma membrane (Fiory et al., 2009).

As mentioned previously, PEA-15 binds the D4 domain of PLD1 with high affinity which catalyses the hydrolysis of phosphatidylcholine to phosphatidic acid (Zhang et al., 2000, Viparelli et al., 2008). PLD activity and the generation of phosphatidic acid have been implicated in signal transduction as well as vesicular membrane trafficking (reviewed in Jones et al. (1999)). Disruption of the interaction between PEA-15 and PLD1 resulted in reduced PKCα activity, similar to control level, and the restoration of insulin sensitivity in skeletal muscle cells in vitro (Viparelli et al., 2008). In addition, insulin sensitivity and secretion were re-established in vivo in mice overexpressing PEA-15 by administration of a recombinant adenoviral vector containing the human D4 cDNA of PLD1 (Cassese et al., 2013). This resulted in overexpression of the D4 domain which interfered with the PEA-15 and PLD1 association (Cassese et al., 2013), suggesting the importance of the interaction between PEA-15 and PLD1 in mediating the detrimental effect on glucose homeostasis. Mice overexpressing PEA-15 selectively in pancreatic β-cells also displayed reduced glucose tolerance in vivo but were found not to be insulin resistant (Miele et al., 2007). Upregulation of PEA-15 affected β-cell function by reducing expression of specific potassium channel subunits in the islets (Miele et al., 2007). These mice also displayed reduced PKCζ activation by glucose and recovery of PKCζ activity resulted in retrieval of basal levels of potassium channel expression as well as insulin secretion (Miele et al., 2007).

In addition to the action on glucose homeostasis, upregulation of PEA-15 has also been implicated in other aspects of type 2 diabetes as well as complications associated with the disease. PEA-15 overexpression increased creatine levels and urine volume in mice and elevated transforming growth factor-β1 (TGF-β1) levels in the kidney and serum (Oriente et al., 2009). Pharmacological inhibition of PLD and PKCβ resulted in a reduction in TGF-β1 and fibronectin production, a component of the extracellular matrix, which could lead to renal dysfunction and kidney damage, a complication associated with type 2 diabetes (Oriente et al., 2009). TGF-β1 can induce autophagy in certain cell types and caused upregulation of PEA-15 in skeletal muscle cells in vitro (Iovino et al., 2012). Atrophic fibres in muscles were also observed in vivo in mice overexpressing PEA-15 which was accompanied with impaired locomotor activity suggesting muscle atrophy in these mice (Iovino et al., 2012).

There are currently no pharmacological tools which directly manipulate PEA-15; however, certain drugs have been shown to potentially mediate their effects through the phosphoprotein. Mice treated with clozapine, an atypical antipsychotic, caused an increase in PEA-15 expression in vivo and exhibited reduced PKCγ activity leading to a reduction in insulin-stimulated glucose uptake (Panariello et al., 2012). In addition, activation of peroxisome proliferator-activated receptor-γ by thiazolidinediones significantly improves insulin sensitivity in type 2 diabetic patients (Nolan et al., 1994, Frias et al., 2000). Pharmacological activation of peroxisome proliferator-activated receptor-γ in skeletal muscle cells reduced transcription of the *pea-15* gene which could be responsible for the beneficial effects witnessed with thiazolidinediones (Ungaro et al., 2012). A more complete understanding of the contribution of PEA-15 in disease progression as well as further identification of the molecules involved in the signalling pathways could lead to the development of novel pharmacological therapies in the treatment of type 2 diabetes.

### 4.2 Phosphoprotein enriched in astrocytes-15 in polycystic ovary syndrome

Similar to type 2 diabetes, PCOS is a heterogeneous endocrine disorder and manifests clinically by hyperandrogenism, chronic anovulation and the presence of multiple cysts on the ovaries in women of reproductive age. Hyperandrogenism or excessive androgen activity is evident by hirsutism or excessive hair growth, acne and female-pattern alopecia in conjunction with chronic anovulation resulting in the prevention of ovulation and oligomenorrhea or infrequent menstruation (Norman et al., 2007). Polycystic ovaries comprise of an elevated number of developing follicles and ovarian volume resulting in menstrual dysfunction and infertility and can also cause obesity and metabolic syndrome in affected women.

There is a well-documented link between PCOS and the risk of developing insulin resistance and type 2 diabetes in premenopausal women which is independent of obesity which is often associated with both disorders (Chang et al., 1983, Dunaif et al., 1989). The altered steroid milieu of PCOS is associated with abnormalities in glucose homeostasis and insulin secretion with about 30 to 40% of women diagnosed with glucose intolerance and 10% with type 2 diabetes (Ehrmann et al., 1999, Savastano et al., 2007). This led to the investigation of PEA-15 expression in PCOS due to its recognised association with type 2 diabetes. PEA-15 expression was significantly higher in both obese and normal weight women with PCOS compared with healthy controls and was also positively correlated with insulin (Savastano et al., 2007). Similar to type 2 diabetic patients, mRNA and protein levels of PEA-15 were found to be elevated in omental adipose tissue of women with PCOS compared to matched controls (Tan et al., 2011). Addition of glucose and insulin to omental adipose tissue explants ex vivo resulted in an upregulation of PEA-15 levels whereas metformin, a routinely used antidiabetic drug, caused a reduction in PEA-15 expression (Tan et al., 2011). This is in agreement with another study which found that after 6 months of treatment with metformin, there was a reduction in PEA-15 protein levels in obese women with PCOS which was also independent of body weight (Savastano et al., 2010). In addition, PCOS is often associated with a deficiency in vitamin D which facilitates intestinal absorption of calcium and phosphorus. Lower levels of serum 25-hydroxyvitamin D, a metabolite produced in the liver by hydroxylation of vitamin D, and an elevated leptin-to-adiponectin ratio, a biomarker of insulin resistance, were correlated with PEA-15 expression in women with PCOS (Savastano et al., 2011). Together, this suggests that an involvement of PEA-15 in PCOS as well as in the insulin resistance, commonly associated with the disease, and by targeting PEA-15 or a component of the signalling pathway by pharmacological intervention could potentially alleviate some of the symptoms of PCOS.

## 5 Phosphoprotein enriched in astrocytes-15 in neurological disorders

As PEA-15 was originally discovered as a protein abundant in astrocytes, its potential role in neurophysiology would seem to be obvious. Although earlier studies have investigated the function of PEA-15 in astrocytes, this relates mostly to cellular signalling effects on proliferation and apoptosis predominantly with relevance to cancer (Renault et al., 2003). Relatively few studies have examined PEA-15 in the context of neurological functions and non-cancer diseases. PEA-15 is widely expressed throughout the brain in a variety of different areas and neuronal cell types (Sharif et al., 2004). Notably, even within the same neuronal cell types, PEA-15 expression varied suggesting the presence of subpopulations. The relevance of this is currently unclear. One study has examined the possible role of PEA-15 in learning (Ramos et al., 2009). PEA-15 null mice displayed impaired learning in a number of different spatial learning tasks including the water maze and odour discrimination and choice. This difference does not appear to be due to changes in brain morphology but is probably the result of changes in ERK1/2 signalling as the differences observed in learning were similar to those observed in mice with impaired ERK1/2 activation (Blum et al., 1999). PEA-15 may therefore play a role in learning and memory via its increased expression in specific areas, such as the hippocampus.

An indication that PEA-15 may have an important role in the brain pathology has recently been revealed by proteomic screens for various neurodegenerative conditions. A recent study examined brains from an Alzheimer's disease transgenic mouse model (Takano et al., 2012). Alzheimer's disease is characterised by the formation of plaques (consisting of β-amyloid peptide) and tangles (made up of tau protein) which are thought to contribute to the impaired neural function and associated memory loss. PEA-15 was one of 20 proteins which was significantly reduced in the hippocampus and cerebral cortex of the Alzheimer's disease transgenic model (Takano et al., 2012). In contrast, a separate study on the neocortex of a different transgenic mouse model of Alzheimer's disease focussed on the neocortical amyloid plaques. In these plaques, PEA-15 expression was increased compared to controls and confirmed in humans by studying amyloid plaques from post-mortem Alzheimer's disease brains (Thomason et al., 2013). These contradictory changes in PEA-15 expression observed in different Alzheimer's models may represent variations in distinct brain regions or neural cell populations as previously observed (Sharif et al., 2004). Using mouse models for different neurodegenerative disorders (including Huntington's disease, the prion disease Scrapie and a model of impaired synaptic transmission), a proteomic study identified PEA-15 as a protein with decreased expression in all these disease models (Zabel et al., 2006). The authors termed this a “nodal” protein indicating that PEA-15 sits at a critical convergence in intracellular signalling (as reviewed above) associated with a range of neurological disorders. As yet no studies have examined the potential role that PEA-15 may play in any of the mentioned neurodegenerative conditions. How, or indeed if, PEA-15 is involved in the pathogenesis of these diseases remains to be determined.

Cerebral ischaemia is a result of an interruption of cerebral blood flow and can occur through infarction in the cerebral arteries (ischaemic stroke). PEA-15 gene expression has been reported to be altered in ischaemic neurons. In an in vivo rat model which involves occlusion of the middle cerebral artery, PEA-15 protein expression was decreased (Sung et al., 2010, Koh, 2012). The decreased expression could be reversed by the anti-oxidant, ferulic acid which may account for some of the beneficial effects of this reagent. This somewhat contradicts in vitro experiments with cultured cortical neurons maintained in a glucose- and oxygen-deprived environment which did not detect any change in PEA-15 gene expression (Yu et al., 2009).

## 6 Phosphoprotein enriched in astrocytes-15 in cardiovascular disease

Considering the intracellular role that PEA-15 has in regulating proliferation and apoptosis, it is perhaps not surprising that, in addition to cancer, this phosphoprotein could be involved in other diseases where similar cellular processes occur. With respect to the cardiovascular system, the principal cell types involved in physiological regulation are endothelial and VSM cells. PEA-15 has a role in both endothelial and VSM cell function (Han et al., 2011, Hunter et al., 2011). Cardiovascular disease, an umbrella term which encompasses a number of pathologies associated with the cardiovascular system, typically results from abnormal regulation of, or damage to, one of these cell types.

In endothelial cells, increased reactive nitrogen species such as peroxynitrite, is associated with endothelial cell death (Otani, 2009). Reactive nitrogen species are produced as a result of ischaemia, for example following stroke or myocardial infarction. Damage to the endothelial cells could prevent or at least slow recovery of the damaged myocardium. A recent study has demonstrated that PEA-15 expression is decreased during ischaemia in vitro as a result of incubation with peroxynitrite and this possibly contributes to endothelial cell death, typically observed following ischaemic injury (Han et al., 2011). Overexpression of phosphorylated PEA-15 prevented this effect suggesting that elevating PEA-15 expression and/or increasing phosphorylation therefore could be a potential therapeutic option in ischaemia.

In atherosclerosis, remodelling of the arterial wall occurs due to two characteristics which are now known to play important pathogenic roles. These are lipid deposition in the intimal space and the infiltration of inflammatory cells. The result of both these processes is the formation of a lipid-containing plaque with an abundance of inflammatory cells. As the plaque grows, the inward remodelling can become unstable and lead to thrombosis and/or plaque rupture which ultimately results in arterial blockage. Inflammation is now regarded as a major aspect of the atherosclerotic process (Hansson & Hermansson, 2011). Initially, monocytes are recruited to the plaque region as it develops and this is followed by T-lymphocytes (Tse et al., 2013). In mature plaques, it is likely that much of the inflammation which drives vessel remodelling and plaque stability is regulated by lymphocytes. The role of PEA-15 in T-lymphocytes has been examined in the PEA-15 null mouse model (Pastorino et al., 2010). Although these mice do not have any specific differences in T-lymphocyte number under non-pathogenic conditions, an inflammatory challenge (in vitro or in vivo) results in an increased T-lymphocyte proliferation. In addition, there is an increased activation leading to enhanced production of IL-2. Although this has not yet been examined in atherosclerotic models, increased T-lymphocyte proliferation and activation could exacerbate the inflammation associated with atherosclerotic plaques. Indeed, activation of T-lymphocytes is increased when present in the plaque compared with those present in the blood (Grivel et al., 2011). It is possible that disease-induced changes in PEA-15 expression could be involved in this increased activation.

In cardiovascular disease, VSM cells in the artery wall modulate towards a proliferative phenotype (Owens et al., 2004). This has been demonstrated both in atherosclerosis and in the vascular injury response to percutaneous coronary intervention such as balloon angioplasty (Marx et al., 2011, Hopkins, 2013). In particular, the restenotic response is predominantly due to VSM cell proliferation and, despite the advent of arterial stents to prevent this, re-occlusion still occurs in ~40–50% of cases. The intracellular pathways leading to the switch in VSM cell phenotype from a fully differentiated contractile cell to a proliferating state are partly delineated. ERK1/2 is activated by growth factor receptors, most notably PDGF-β receptor tyrosine kinase which binds PDGF-BB and this leads directly to the phosphorylation of Elk-1 in the nucleus (Kawai-Kowase & Owens, 2007). Elk-1 interacts with another transcription factor, serum response factor, and represses VSM cell marker genes such as smooth muscle α-actin and smooth muscle myosin heavy chain in addition to activating genes associated with proliferation (Kawai-Kowase & Owens, 2007). We have recently demonstrated that PEA-15 phosphorylation is required for the activation of Elk-1 in human coronary artery VSM cells (Hunter et al., 2011). Preventing PEA-15 phosphorylation by either siRNA knockdown of phospholipase Cγ (to remove the upstream phosphorylation pathway) or overexpressing nonphosphorylatable PEA-15 mutants, PDGF-BB stimulation failed to fully phosphorylate Elk-1 and the subsequent repression of the smooth muscle α-actin gene. Furthermore, decreasing PEA-15 expression led to VSM cell proliferation whereas overexpressing wild type PEA-15 inhibited ERK1/2 translocation to the nucleus (Hunter et al., 2011). Although further research is required to determine the role in vascular diseases where VSM cell proliferation has a prominent role, such as restenosis, maintaining PEA-15 expression in VSM cells could be vasculoprotective.

## 7 Conclusions

PEA-15 is undoubtedly a pivotal and important protein in regulating major intracellular processes including proliferation and apoptosis. As PEA-15 is expressed in many different cell types, the ultimate physiological effects of this regulation are tissue/organ-dependent. It is therefore not surprising that PEA-15 has been implicated in playing a key physiological role in many diverse biological systems including the brain, ovaries, skeletal muscle, blood vessels and immune cells. From many studies, it is now also apparent that altered PEA-15 regulation and/or expression may be associated with various disease states. Evidence is now emerging for a potential involvement of PEA-15 in cancer, type 2 diabetes, PCOS, Alzheimer's disease and cardiovascular disease. In some of these diseases, particularly cancer and type 2 diabetes, the links to PEA-15 pathology are becoming clearer. Through these studies, a potential therapeutic target has been partially characterised. Future studies should provide a more detailed picture for the roles of PEA-15 in disease states, possibly adding to the list of diseases potentially affected by PEA-15 regulation. This research will start to identify approaches to manipulating PEA-15-induced pathological effects in vivo and the route leading to novel therapies should appear on the horizon.

## Funding sources

FHG and GFN are supported by a 10.13039/501100000925National Health and Medical Research Council (grant MR/K012789) research grant. Work in the laboratory has previously been funded by the 10.13039/501100000274British Heart Foundation and the 10.13039/100004440Wellcome Trust.

## Conflict of interest statement

The authors declare that there are no conflicts of interest.
